# Anti‐Inflammatory Treatment of Subarachnoid Hemorrhage by Self‐Assembled Silymarin Nanoparticles

**DOI:** 10.1002/smsc.202400322

**Published:** 2025-02-03

**Authors:** Yong Li, Youdong Zhou, Yinqiu Tan, Gang Deng

**Affiliations:** ^1^ Department of Neurosurgery Renmin Hospital of Wuhan University No. 99 Zhangzhidong Road Wuhan Hubei 430060 China; ^2^ Yichang Central People's Hospital No. 183 Yiling Avenue Yichang City Hubei Province 443000 China

**Keywords:** early brain injuries, nanoparticles, neuroinflammation, silymarin, subarachnoid hemorrhage

## Abstract

Subarachnoid hemorrhage (SAH) is a common hemorrhagic cerebrovascular disease with high disability rate and high mortality. Early brain injury (EBI) is the main cause of high mortality and delayed neurological dysfunction in patients with SAH. Neuroinflammation is the important pathological processes of EBI.We prepared Silymarin nanoparticles (SIM NPs) through the solvent precipitation method and investigated their role in combating EBI following SAH in mice. We found that SIM NPs with a diameter of 150 nm have the strongest ability to cross the blood‐brain barrier. SIM nanoparticles are spherical and contain irregular particles inside, which may be composed mainly of silibinin and assembled through hydrogen bonding. Further in vivo experiments showed that SIM NPs improved short‐term neurological dysfunction in SAH mice, reduced cortical neural damage, and reduced EBI inflammation through the Nrf2/STING pathway. Finally, water maze experiments showed that SIM NPs can improve long‐term memory and learning ability in SAH mice. Based on the above results, we conclude that silymarin nanoparticles can reduce EBI after SAH by inhibiting the Nrf2/STING pathway, inhibiting neuroinflammation and M1 polarization of microglia.

## Graphical abstract

Herein, nanoparticles self‐assembled from silymarin have been developed and they are applied in the treatment of neuroinflammation following subarachnoid hemorrhage. The findings reveal that these silymarin nanoparticles can inhibit neuroinflammation through the stimulator of interferon gene/interferon regulatory factor 3 pathway.

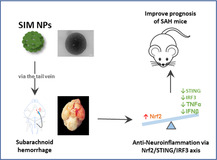

## 1 Introduction

Subarachnoid hemorrhage (SAH) is a common hemorrhagic cerebrovascular disease in neurosurgery, accounting for 5–10% of all strokes, of which 85% are caused by ruptured intracranial aneurysms.^[^ ^1^ ^]^ Although its incidence is relatively low, it has the characteristics of high disability rate and high mortality.^[^ ^2^ ^]^ Currently, the main treatment for SAH is surgical treatment aimed at the underlying cause, including interventional embolization of intracranial aneurysms and open craniotomy aneurysm clipping.^[^ ^3^ ^]^ However, despite significant improvements in the prognosis of patients with SAH, most patients experience some degree of neurological dysfunction that can seriously affect their quality of life.

More and more studies have shown that early brain injury (EBI) is the main cause of high mortality and delayed neurological dysfunction in patients with SAH. EBI refers to brain injury that occurs within 72 h after the onset of SAH.^[^ ^4^ ^]^ Neuroinflammation is one of the important pathological processes of EBI. The characteristics of neuroinflammation are the activation of immune cells and the production of proinflammatory cytokines. Microglia are resident immune cells in the brain and are the main mediators of neuroinflammation. M1 microglia are usually activated by immune receptors including toll‐like receptors and nucleotide‐binding oligomerization domain‐like receptors, which can secrete proinflammatory cytokines and chemokines such as IL‐1β, IL‐6, tumor necrosis factor (TNF), and interferon (IFN) to recognize pathogens and respond to them.^[^ ^5^, ^6^, ^7^ ^]^ This is the basic reaction to maintain homeostasis in the central nervous system. However, long‐term or excessive neuroinflammation may be harmful and can lead to neuronal damage and neurological dysfunction.^[^ ^8^ ^]^ Previous studies have confirmed that microglia are widely involved in the occurrence and development of neuroinflammation in EBI. Recent studies have shown that regulating the signaling pathways involved in microglia activation and neuroinflammation can improve the prognosis of neurological diseases such as SAH.^[^ ^9^, ^10^, ^11^ ^]^

Stimulator of interferon genes (STING) is a protein involved in innate immune responses that forms a dimer and moves from the endoplasmic reticulum to perinuclear structures such as the Golgi apparatus. STING is a cytoplasmic DNA sensor that is widely distributed in mammalian immune cells, including DC cells, T cells, and macrophages, and functions as an important mediator of innate immune responses and inflammation.^[^ ^12^ ^]^ When stimulated by exogenous DNA, STING recruits Tbk1 and promotes phosphorylation of interferon regulatory factor 3 (IRF3), which then enters the nucleus and activates transcription of genes encoding interferons and other cytokines.^[^ ^13^ ^]^ In the nervous system, STING is mainly distributed in microglia, where its expression is significantly upregulated after SAH, leading to brain edema and neuronal damage.^[^ ^14^ ^]^ Nuclear factor erythroid 2‐related factor 2 (Nrf2) is a main transcription factor that maintains redox balance and combats oxidative stress and inflammation in cells. Many studies have shown that activating Nrf2 can reduce STING activation and inhibit inflammation.^[^ ^15^ ^]^

Herbal has been widely used in traditional medicine around the world. Many active ingredients in herbs have anti‐inflammatory and antioxidant effects.^[^ ^16^ ^]^ However, most compounds in these herbs have limited ability to penetrate the brain.^[^ ^17^ ^]^ Silymarin (SIM) is a mixture extracted from the fruit of the natural plant milk thistle, which has significant antioxidant, anti‐inflammatory, and proapoptotic properties, as well as multiple biological and pharmacological activities, such as liver protection, antidiabetes, anticancer, heart protection, light protection, and immune modulation.^[^ ^18^, ^19^, ^20^, ^21^, ^22^, ^23^, ^24^, ^25^ ^]^ In the Ischemic stroke, the anti‐inflammatory effect of SIM is primarily attributed to its inhibition of the nuclear translocation/activation of NF‐κB, which subsequently leads to a reduction in inflammatory cytokines. These events impede the accumulation of inflammatory cells, followed by decreased activities of iNOS and MPO.^[^ ^26^ ^]^ Additionally, SIM has been reported to inhibit the activities of 5‐lipoxygenase and COX, thereby suppressing the production of leukotrienes and prostaglandins.^[^ ^27^ ^]^ Additional reports indicate that SIM can ameliorate depressive/anxiety‐like behaviors in mouse models of Parkinson's disease by inhibiting the STING‐IRF3 pathway.^[^ ^28^ ^]^ However, its role in SAH is currently unknown.

Recently, using natural biomass as a reactant or structure to build functional nanostructures has become a hot research topic.^[^ ^29^, ^30^, ^31^, ^32^ ^]^ In this experiment, we explored the potential of using SIM to construct functional nanostructures and successfully constructed self‐assembled SIM nanoparticles (NPs). By controlling the size of these NPs, we enabled their enrichment in SAH tissue. In a mouse model of SAH, self‐assembled SIM NPs demonstrated effective penetration of the blood–brain barrier and exhibited STING inhibition, possessing the potential to effectively regulate neuroinflammation after SAH. By mixing multiple components to construct coassembled NPs, this provides a new strategy for utilizing natural biomass to construct functional nanostructures.

## 2 Experimental Section

### 2.1 Materials and Antibodies

SIM (purity: 80%) and silybin (SIL) (purity: 98%) were purchased from Shanghai Aladdin Biochemical Technology Co., Ltd., China. Antibodies used included the following: anti‐Nrf2 [12721T, Cell Signaling Technology (CST), USA], anti‐STING [13647S, CST, USA], anti‐Phospho‐IRF3 [4947S, CST, USA], anti‐Phospho‐NF‐κB p65 [3033S, CST, USA], anti‐beta actin [GB15001, Servicebio, China], and anti‐beta actin [GB15140, Servicebio, China].

### 2.2 Animals

Male C57BL/6J mice weighing 20–22 g were purchased from China Slake Experimental Animal Co., Ltd. and were raised at room temperature (22 ± 1 °C) and 12 h day night circulation. Animals had free access to food and water. All animal procedures were performed in compliance with international guidelines on the ethical use of animals and were approved by the Laboratory Animal Ethics Committee of Renmin Hospital of Wuhan University (WDRY20210305A).

### 2.3 SAH Model

As mentioned earlier, a SAH model was established through intravascular perforation under 2% isoflurane anesthesia. During the surgery, the animal's body temperature was maintained at 36.5 °C. After satisfactory anesthesia, the left internal carotid artery and external carotid artery were carefully separated. The common carotid artery and internal carotid artery were temporarily blocked with two small vascular clamps. SAH was caused by puncturing the left middle cerebral artery using silk thread (Doccol Corp.). The sham‐operated mice underwent the same surgical procedure, but did not burst.^[^ ^33^ ^]^ When the mice lost more than 20% of their weight or experienced persistent uncontrollable pain or convulsions, we euthanized the mice. At the end of study, the animals were sacrificed with cervical dislocation method following anesthesia with 3% isoflurane.

### 2.4 Study Design

**Figure 1:**
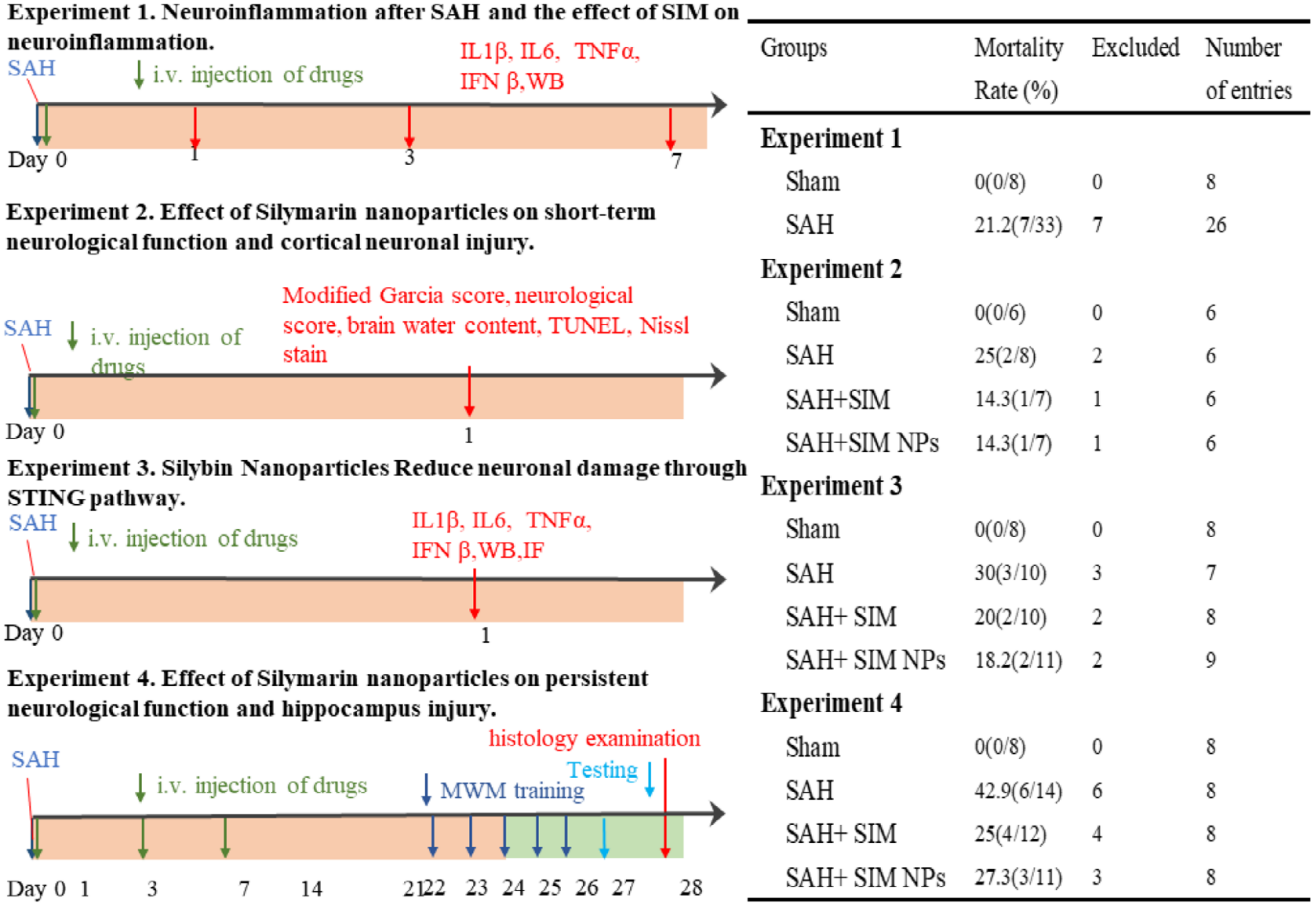
Experimental designs of the study.

As shown in **Figure** **1**, mice were randomly allocated to four separate experiments. Mice that died within 24 h or those with mild SAH (SAH grading score < 8) were excluded from the current study. After the surgery, the mice were randomly divided into sham, SAH, SAH + SIM, and SAH + SIM NPs groups. Based on past studies, we had decided to use a dosage of 100 mg kg^−1^. To determine the protective effect of SIM NPs on EBI after SAH, SIM NPs (dissolved in PBS) or SIM (dissolved in 10% DMSO + 40% PEG400 + 5% Tween 80 + 45% saline) were injected into the mice via the tail vein 1 h after the surgery, while the sham and SAH groups received the same dose of solubilizer. 24 h after the successful establishment of the model, the mice were subjected to a neurological function score, followed by relevant pharmacological experiment. To assess the protective effect of SIM NPs on persistent neurological deficits after SAH, a single dose was administered at 1 h, followed by daily administrations for seven consecutive days.

### 2.5 Cerebral Blood Flow Measurement

Mice were anesthetized with 2% isoflurane. The scalp was incised and fixed on the detection platform. The focus under the scanning probe was adjusted, and a laser speckle blood flow was imaged using imaging system (Simopto, Wuhan, China).

### 2.6 SAH Grade

According to the documentation method, two experimenters independently scored, and the average value was recorded as the final score.^[^ ^34^ ^]^ The ventral part of the mouse brain was divided into six parts, and 0–3 subsystems were measured according to the volume of blood clot. The sum of the six parts was calculated as a total score (0–18). The mice with SAH score lower than 7 or higher than 15 will not be subject to subsequent experimental analysis.

### 2.7 Modified Garcia Score

The short‐term results were measured by the modified Garcia score (3–18 points scoring system).^[^ ^35^ ^]^ The modified Garcia score is composed of six parts: spontaneous activity, spontaneous limb movement, forelimb extension, tactile sensation, body proprioception, and climbing ability. This scoring was conducted using a double‐blind design, meaning that data collection and analysis were performed by a third party unaware of the mice's grouping information.

### 2.8 Neurological Function Assessment

Seventy‐two hours after reperfusion, the neurological function assessments of all mice were performed using the Longa scoring method. The rating scale was as follows: 0 point, no neurological deficits; 1 point, inability to fully extend the contralateral forepaw; 2 points, circling to the contralateral side; 3 points, falling to the contralateral side; and 4 points, inability to walk spontaneously and with a depressed level of consciousness. Similarly, a double‐blind design was adopted for this scoring to ensure fairness and objectivity in the evaluation process. Data collection and analysis were performed by a third party unaware of the mice's grouping information.

### 2.9 Synthesis of NPs

10 mg of SIM was dissolved in 1 mL of dichloromethane, centrifuged to remove insolubles, and the solution was added dropwise to a shaken 2.5% polyvinyl alcohol (PVA) solution. The obtained emulsion was sonicated on ice, poured into 30 mL of 0.3% PVA aqueous solution, and stirred overnight. The liquid was collected and centrifuged at 18 000 rpm for 30 min to enrich the product. The precipitate was resuspended in distilled water and centrifuged again to remove all PVA from the liquid. Finally, the precipitate was lyophilized and stored. The particle size of the NPs was controlled by controlling the ultrasonic intensity and PVA volume.

### 2.10 Dynamic Light Scattering

SIM NPs were diluted to 1 mg mL^−1^ aqueous solution. The hydration diameter and zeta potential were measured by dynamic light scatterer (Zetasizer Nano ZSP, Malvern instruments Ltd., UK).

### 2.11 Nissl Staining

Nissl staining was conducted to demonstrate the number of normal neuron in cortex and hippocampus as previously reported. The frozen brain slides were immersed with 0.5% cresyl violet solution and dehydrated with 100% alcohol. After sealed with neutral balsam, the slides were observed under a light microscope by a blinded investigator.

### 2.12 TUNEL Staining

After the slice was dehydrated, it was put in the TUNEL reaction buffer to incubate 1 h at 37 °C [12156792910, Roche, Switzerland]. The confocal microscope was used to observe the slice and the apoptotic neurons are red.

### 2.13 Inflammatory Cytokines Detection

Brain tissue was homogenized with 10 times its weight of PBS. The homogenate was centrifuged at 12 000 g for 5 min, and the supernatant was collected. After adding the sample, the detection antibody was added, followed by SA‐HRP. Tetramethylbenzidine was used for color development. IL1β (GEM0002, Servicebio, China), IL6 (GEM0001, Servicebio, China), IFNβ (GEM0018, Servicebio, China), and TNF‐α (GEM0004, Servicebio, China) were detected.

### 2.14 Rotarod Test

Before the experiment, the mice received training to adapt to the rotating rod. The mice were placed on a treadmill (Media Associates, St Albans, VT). When the mouse moved on the rod, the rod with a diameter of 32 mm was raised to 16 cm above the laboratory workbench and raised to 10 rpm within 300 s to evaluate the average fall latency. Three experiments were conducted with at least a 5 min interval between each trial to evaluate the average fall latency. During the rotational test, both the operator and the data analyzer were blinded to the mice's grouping status, employing a double‐blind method to prevent potential bias.

### 2.15 Morris Water Maze

We used the Morris water maze (MWM) test to evaluate the learning and memory functions of mice. During the 21–26 days, the mice were trained to locate the underwater platform within 60 s. If the mice failed to find the platform, they would be guided to it and allowed to stay on the platform for an additional 15 s. On the 27th day, the underwater platform was removed, and the mice were placed in any quadrant of the water maze and observed for their movement trajectory within 60 s. A double‐blind approach was implemented, where the evaluator was unaware of the specific grouping (sham, SAH, SAH + SIM, SAH + SIM NPs) of the mice during scoring. All mice were identified through coded labels to conceal their experimental conditions.

### 2.16 Statistical Analysis and Power Calculations

This study estimates the sample size for comparing the mean of a sample with the population mean, where the population mean belongs to the SAH group, and the sample group is the SAH + SIM NPs group. The observed outcome indicator is the modified Garcia score. According to the literature review and preliminary experimental results, the mean modified Garcia score of the SAH group is 10.2 ± 2.2 points, and it is anticipated that the SAH + SIM NPs group will have an average increase of 1.8 points in the score. Assuming a two‐sided *α* = 0.05 and *β* = 0.2, the PASS 15 software calculated the required sample size to be nine cases for the treatment group and eight cases for the control group. However, considering a 25% mortality rate due to surgery, the final minimum sample size for each group should be at least 13 cases. All experiments were conducted a minimum of three times, and the data were presented as mean ± standard deviation (SD). The significance of differences between two groups was evaluated by performing a two‐tailed unpaired Student's *t*‐test using of SPSS software version 24. For comparisons involving multiple groups, a one‐way analysis of variance was conducted, followed by a Tukey post hoc test. The normality of data was assessed using the Shapiro–Wilk test, and the data with non‐normal distribution were evaluated using the Mann–Whitney *U* test. All statistical charts were created using GraphPad Prism 8.0.

## 3 Result

### 3.1 The Therapeutic Effect of SIM on Neuroinflammation after SAH

**Figure 2:**
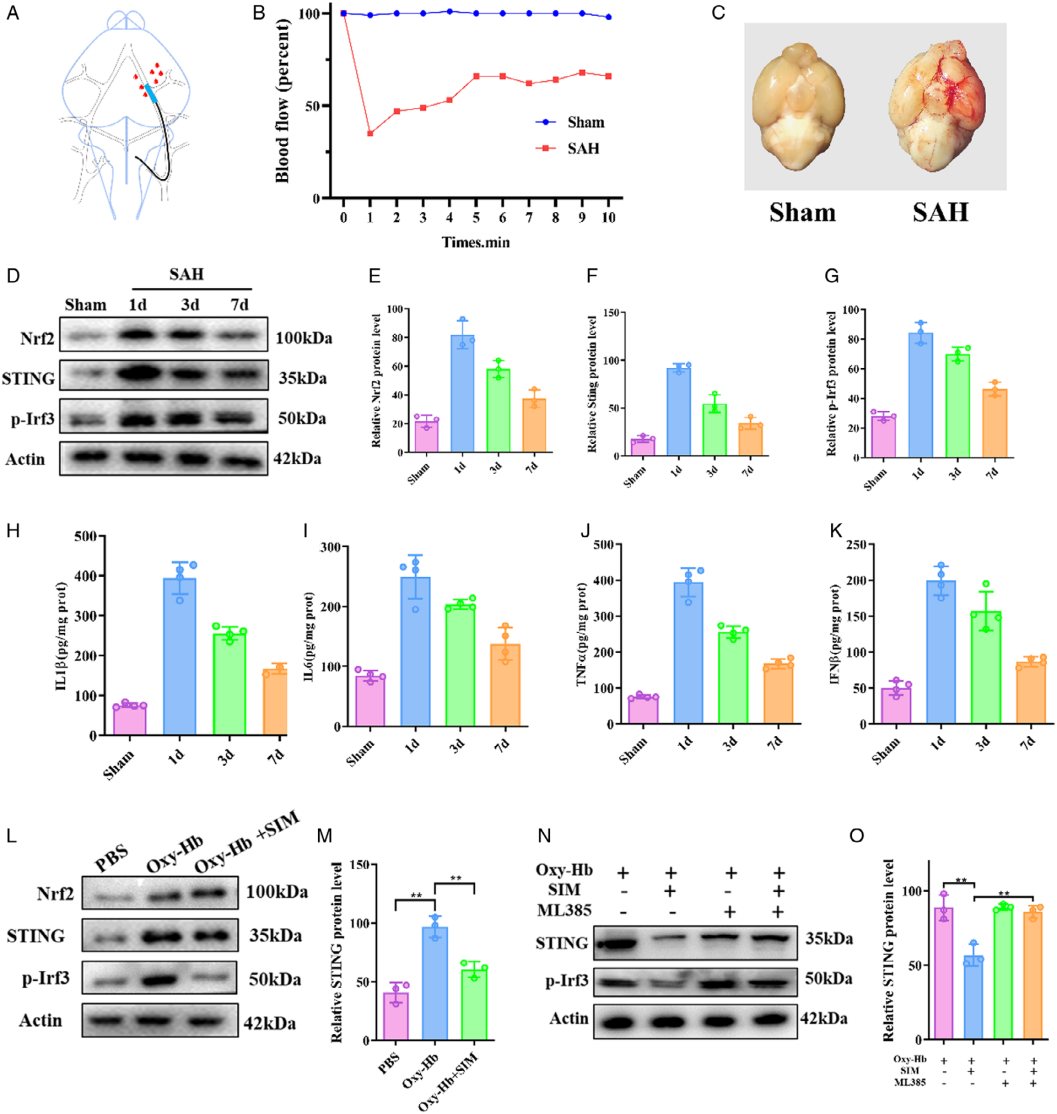
Neuroinflammation after SAH and the effect of SIM on neuroinflammation. A) Schematic diagram of modeling. B) Ultrasonic Doppler monitoring the changes in cerebral blood flow from the left MCA blood supply area. C) Representative images of the SAH region. Scale bar = 100 μm. D‐G) Representative western blotting images and quantification of Nrf2, STING, and *p*‐Irf3 expression in the temporal cortex at 1, 3, and 7 days after SAH (*n* = 4). H–K) Relative IL1β, IL6, TNF‐α, and IFNβ level in the temporal cortex at 1, 3, and 7 days after SAH (*n* = 4). **P < 0.01; ***P < 0.001. L,M) Representative western blotting images of Nrf2, STING, and *p*‐Irf3 in BV2 cells after 20 μg mL^−1^ SIM treatment. N,O) Representative western blotting images of Nrf2, STING, and *p*‐Irf3 in BV2 cells after 20 μg mL^−1^ SIM and ML385 treatment.

As shown in **Figure** **2**A, we manufactured SAH mice model using intravascular puncture. By dynamically monitoring cerebral blood flow, we found that at the moment of puncturing blood vessels, the cerebral blood flow of the middle cerebral artery decreased, and after removing the thread plug, the blood flow recovered, which can be used to determine the success of modeling (Figure 2B). Then we euthanized the SAH mice and dissected their brains to evaluate the degree of SAH. We found diffuse SAH around the anterior cerebral artery, middle cerebral artery, basal cistern, and some temporal lobe cortex in mice (Figure 2C).

To investigate the activation of STING after SAH, we subjected the mice to SAH and analyzed the brain tissue at 1, 3, and 7 days after modeling. We found that the protein expression levels of Nrf2, STING, and transcription factor IRF3 downstream of STING increased at 1 day after SAH, and then gradually decreased (Figure 2D–G). Further detection of the concentrations of IL1β, IL6, TNF‐α, and IFNβ in brain tissue revealed an increase in inflammatory cytokine levels after 1 day of SAH, followed by a gradual decrease (Figure 2H–K). Therefore, we concluded that STING induced neuroinflammation reached the peak 1 day after SAH, so we conducted subsequent experiments at 1 day after SAH. We constructed an in vitro model of SAH by adding oxy‐Hb to mouse microglial cell line BV2 cells to simulate the hypoxia and hemoglobin stimulation environment after SAH. As shown in Figure 1L, after modeling, the levels of Nrf2, STING, and IRF3 proteins increased. The addition of SIM resulted in higher levels of Nrf2, while STING and IRF3 levels were inhibited (Figure 2L,M and S1, Supporting Information). Furthermore, by adding the inhibitor of Nrf2, ML385, it was found that the inhibition of SIM on STING and its downstream IRF3 was weakened (Figure 2N,O and S2, Supporting Information). Therefore, we speculate that SIM can regulate the STING pathway through Nrf2 and achieve anti‐inflammatory effects.

### 3.2 Synthesis and Characterization of SIM NPs

**Figure 3:**
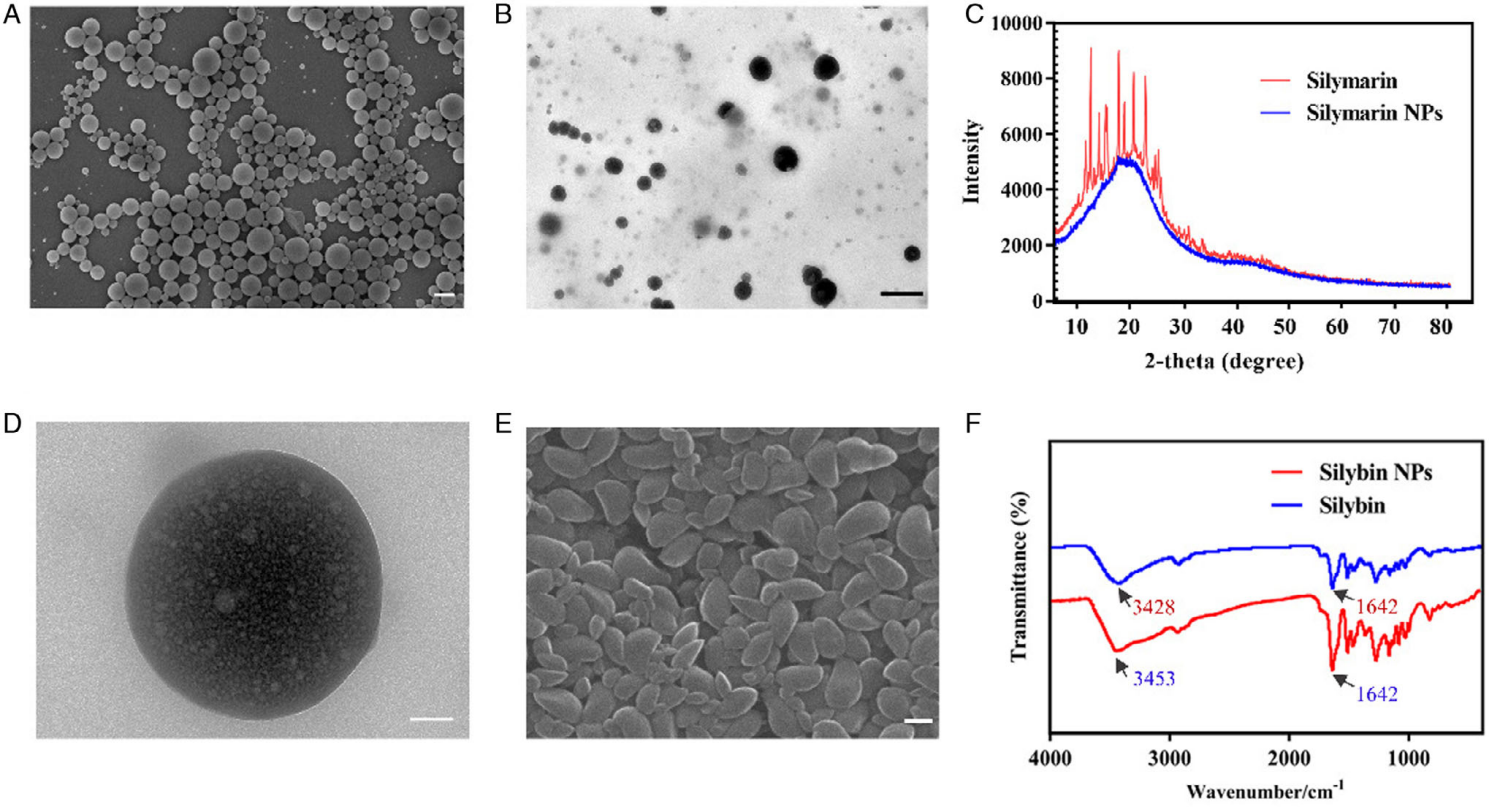
Synthesis and characterization of SIM NPs. A) Representative scanning electron microscope (SEM) image of SIM NPs. Scale bar: 200 nm. B) Representative TEM image of SIM NPs. Scale bar: 50 nm. C) XRD of SIM NPs. D). Representative TEM image of SIM NPs. Scale bar: 50 nm. E) Representative SEM image of SIL NPs. Scale bar: 200 nm. F) Fourier transform infrared spectrometry of SIL NPs.

Previous studies have shown that pentacyclic triterpenoid compounds form self‐assembled NPs through hydrogen bonding interactions, but it is currently unknown whether there are other forms of self‐assembly. SIM is a multicomponent mixture mainly composed of SIL. In this experiment, solvent evaporation method was used to prepare NPs of SIM. As shown in **Figure** **3**A,B, the SIM NPs exhibit irregular spherical shapes of varying sizes, while the SIM NPs have a solid structure. Through X‐ray diffraction (XRD), we found that the SIM NPs exhibit a single peak and are not nanocrystals (Figure 3C). Through LC‐MS, we found that the composition of SIM NPs is basically consistent with that of SIL, isosilybin, silydianin, and silychristin (Figure S3, Supporting Information). The proportion of SIL in SIM NP is 60.2%, which is basically consistent with the content of SIL in SIM. To investigate the formation mechanism of SIM NPs, we found through high‐magnification transmission electron microscope (TEM) that the core of SIM NPs is rich in low‐density particles (Figure 3D). Therefore, we speculate that the formation mechanism of SIM NPs is to self‐assemble a few elements such as SIM and encapsulate other components. Due to the fact that SIL is the main component of SIM NPs, we prepared SIL NPs using solvent evaporation method. We found that SIL NPs were spindle‐shaped (Figure 3E) and studied the intermolecular interactions of SIM through infrared spectroscopy. We found that, compared with free SIL, the O—H in SIL NPs showed redshift (3428 vs 3453), while the vibration of C=O showed no significant change (1642 vs 1642), indicating that intermolecular hydrogen bonds were formed based on O—H in SIL NPs (Figure 3F). Therefore, we finally speculated that SIM NPs were self‐assembled based on SIL through intermolecular hydrogen bonds. This coassembly method was more similar to the stacking of finer spheres rather than the vesicular structure of traditional pentacyclic triterpenes. And the doping of its isomers ultimately led to the generation of solid spherical structures.

### 3.3 Promoting Drug Delivery to the Brain through Size Control

**Figure 4:**
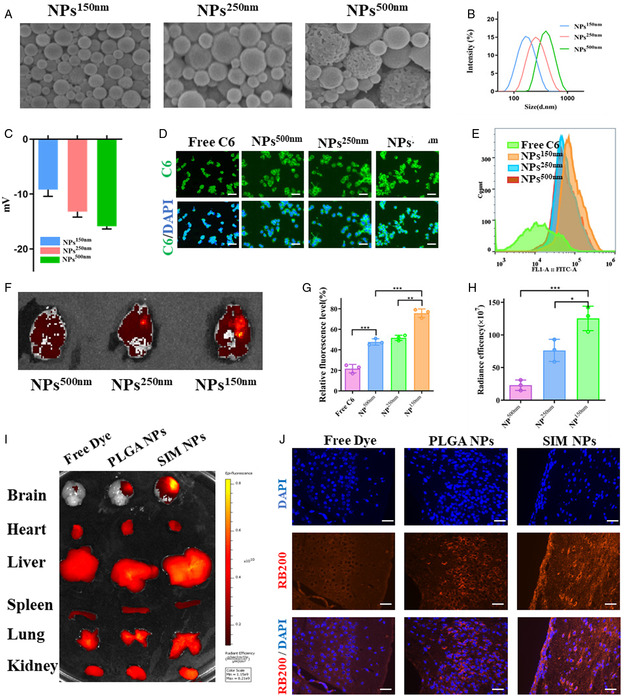
Promoting drug delivery to the brain through size control. A) SEM analysis of the morphological changes of the NPs^150 nm^, NPs^250 nm^, and NPs^500 nm^. Scale bar: 200 nm. B) Dynamic light scattering (DLS) analysis of the NPs^150 nm^, NPs^250 nm^, and NPs^500 nm^ hydrodynamic diameters. C) DLS analysis of the NPs^150 nm^, NPs^250 nm^, and NPs^500 nm^ ζ potentials. D,E,G) Confocal images (D), flow cytometry (E), and quantification analysis (G) of cell uptake of BV2 cells after incubation with different formulations. Scale bar = 20 μm. F,H) Representative image and quantification of the NP signals in mouse brains. I) Representative images of the NPs in the brain, heart, liver, spleen, lung, and kidney of mice receiving the indicated treatment. J) Representative images of the NPs in the regions of SAH brain. Scale bar = 50 μm.

Numerous studies have shown that the size of NPs is closely related to drug delivery efficiency. In order to further analyze the optimal NP size, we controlled the size of SIM NPs by controlling ultrasound intensity and water oil ratio, and manufactured NPs with particle sizes of 150, 250, and 500 nm, respectively (**Figure** **4**A,B). Their zeta potentials are −9.7, −13.5, and −15.2 mV, respectively (Figure 4C), indicating that all three sizes of NPs have considerable stability in aqueous solutions. We first evaluate the cellular uptake efficiency of NPs at 150, 250, and 500 nm. We constructed SIM‐C6 NPs using SIM‐loaded fluorescent dye coumarin 6. Given the lack of spontaneous fluorescence in SIM and cells themselves, any observed fluorescence signal can be attributed to encapsulated coumarin 6. We standardized the drug concentrations of all groups based on fluorescence intensity to ensure consistent dye content in each group. We introduced SIM‐C6 NPs into the culture medium of BV2 cells, incubated them for 20 min, and then observed the results through fluorescence microscopy and flow cytometry (Figure 4D,E,G). The results indicate that when the particle size is 150 nm, the NPs can be more effectively absorbed by cells which may be attributed to their smaller particle size and lower zeta potential. We further investigated the uptake of SIM NPs with different particle sizes in in vivo experiments. RB200 is another fluorescent dye, and due to its long emission wavelength, we chose it as the in vivo tracer dye. We injected SIM‐RB200 NPs into SAH mice through the tail vein, euthanized the mice 24 h later, and captured their brains for IVIS imaging. The results showed that NPs with a size of 150 nm accumulated the most in SAH tissue (Figure 4F,H).

We then compared SIM with the classic nanocarrier poly‐lactic‐co‐glycolic acid (PLGA) to study its distribution in different organs in vivo. We injected SIM‐RB200 NPs and PLGA NPs loaded with RB200 into SAH mice through the tail vein and took mouse organs for IVIS imaging 24 h later. The results indicate that free dyes are mainly absorbed by the liver and have limited pathways to enter brain tissue through the blood–brain barrier. On the contrary, PLGA showed effective enrichment in the SAH injury area, while SIM NPs were further enriched in SAH brain tissue (Figure 4I). Subsequently, confocal observation of brain tissue slices revealed a large number of intact fluorescent particles around the SIM NPs group cells. This emphasizes the SAH tissue targeting ability of SIM NPs (Figure 4J). The specific mechanism of SIM NPs targeting brain tum tissue remains to be further studied. We speculate that SIM NPs can directly enter brain tissue through the open paravascular spaces and, due to the lack of means to expel NPs from the edematous brain tissue, further aggregate around the edematous brain tissue. Therefore, the targeting ability of SIM NPs is primarily determined by their capacity to cross the blood–brain barrier and their release capacity in edematous brain tissue with an acidic environment. In other words, SIM NPs with smaller particle sizes and higher potentials exhibit stronger targeting capabilities. In summary, these findings emphasize that compared to PLGA, SIM NPs have increased bioavailability and enhanced targeting ability toward SAH brain tissue.

### 3.4 Effect of SIM NPs on Short‐Term Neurological Function and Cortical Neuronal Injury

To further apply SIM NPs in mice, we tested the toxicity of SIM NPs. Our experiments revealed that the IC_50_ of SIM NPs on BV2 cells was 363 μg mL^−1^ (Figure S4A, Supporting Information). To further evaluate the safety of SIM NPs, we intravenously injected 100 mg kg^−1^ of SIM NPs into normal mice and monitored their body weight. Compared with the control group, no mice in the SIM NPs group died during the experiment, and there were no significant changes in body weight (Figure S4B, Supporting Information). Additionally, we performed hematoxylin‐eosin staining on the livers and kidneys of mice in both groups and observed no apparent tissue toxicity associated with SIM NPs (Figure S4C, Supporting Information). These findings suggest that SIM NPs exhibit high biosafety and can be used for intravenous therapeutic purposes.

**Figure 5:**
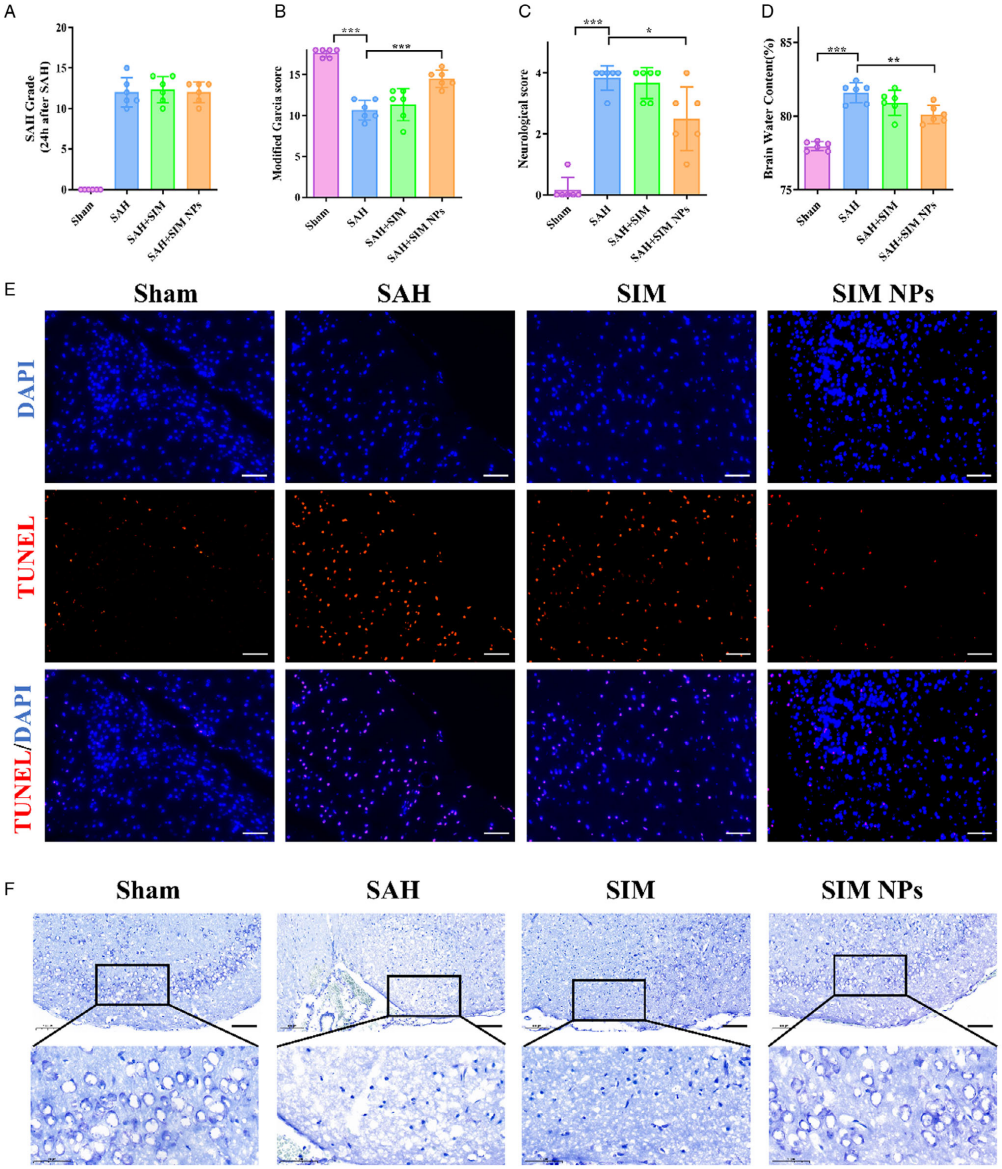
Effect of SIM NPs on short‐term neurological function and cortical neuronal injury. A) Quantification of SAH grade. B,C) Quantification of neurological function with modified Garcia score systems (B) and neurological score systems (C) 24 h after SIM NPs treatment (*n* = 6). *P < 0.05; **P < 0.01; ***P < 0.001. D) Quantification of brain water content 24 h after SIM NPs treatment (*n* = 6). *P < 0.05; **P < 0.01; ***P < 0.001. E) Representative images of TUNEL staining in temporal cortex 24 h after SIM NPs treatment (*n* = 3). *P < 0.05; **P < 0.01; ***P < 0.001. Scale bar = 50 μm. F) Representative images of Nissl staining in temporal cortex 24 h after SIM NPs treatment (*n* = 3). *P < 0.05; **P < 0.01; ***P < 0.001. Scale bar = 100 μm.

We further evaluate the therapeutic potential of SIM NPs in SAH mouse models. After successfully establishing the model, the mice were immediately administered via tail vein at a dose of 0.1 mg g^−1^. 24 h later, the mice were euthanized to collect brain tissue for assessment of the severity of SAH before neurological deficit scores were assigned. The SAH score analysis showed no significant differences between the experimental groups (Fig**ure** **5**A). Using the improved Garcia score and neurological score, we observed that mice exhibited significant neurological dysfunction 24 h after SAH induction. SIM did not improve neurological dysfunction in SAH mice, while SIM NPs significantly improved neurological dysfunction in SAH mice (Figure 5B,C). This may be due to the inability of SIM to cross the blood–brain barrier, resulting in weaker therapeutic effects, while SIM NPs can provide better therapeutic effects. Brain edema is a result of ischemic hypoxia of brain tissue following SAH and is an important independent risk factor for poor prognosis after SAH. Compared with the sham group, the brain tissue water content was significantly increased in the SAH group at 24 h. Notably, application of SIM NPs significantly reduced brain tissue water content (Figure 5D).

TUNEL‐positive staining indicates severe cell damage, while well‐developed rough endoplasmic reticulum and free ribosomes, which form Nissl bodies, reflect neuronal activity. Using TUNEL and Nissl staining on brain tissue samples, it was evident that SIM led to a decrease in the proportion of TUNEL‐positive cells and an increase in Nissl body abundance compared with SAH group mice (Figure 5E,F). This highlights the neuroprotective potential of SIM NPs. Notably, the superior effect of SIM NP compared with SIM may be attributed to its enhanced target affinity for SAH‐affected tissues, resulting in a higher concentration of NPs in the local tissue environment.

### 3.5 SIM NPs Reduce Neuronal Damage through STING Pathway

**Figure 6:**
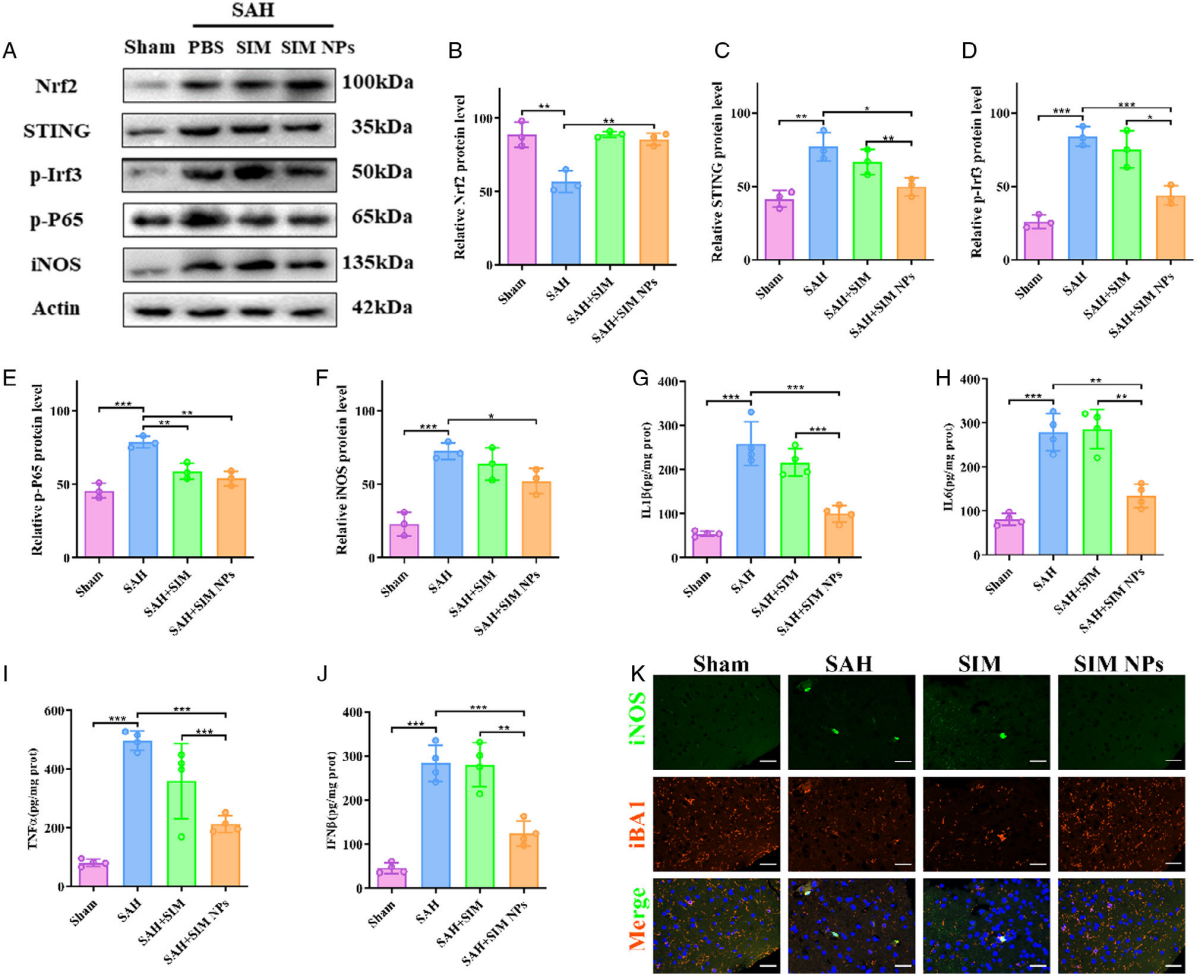
SIM NPs reduce neuronal damage through STING pathway. A–F) Representative western blotting images and quantitative analyses of STING pathway after SIM NPs treatment in vivo (*n* = 3). *P < 0.05; **P < 0.01; ***P < 0.001. G–J) IL1β, IL6, TNF‐α, and IFNβ level 24 h after SIM NPs treatment in vivo (*n* = 4). *P < 0.05; **P < 0.01; ***P < 0.001. (K).

We further extracted proteins from the temporal lobe cortex affected by blood stimulation for subsequent Western blot analysis. Similar to the observations at the cellular level, our results showed that SIM enhanced the expression of Nrf‐2 in brain tissue after SAH, reduced the phosphorylation of STING pathway protein IRF3, and decreased the expression of M1‐type glial cell marker iNOS after SAH (**Figure** **6**A–F). Compared with SIM, the effect of SIM NPs was more pronounced. This enhancement can be attributed to the enhanced tissue‐specific targeting of SIM NPs.

We further examined the changes in the levels of early inflammatory factors. The experimental results showed that the concentrations of early inflammatory factors and iron ions significantly increased 24 h after SAH. Although SIM alone did not produce substantial improvement, the administration of SIM NPs effectively reduced the secretion of inflammatory factors (Figure 6G–J). Immunofluorescence staining showed that after treatment with SIM NPs, the M1 polarization of microglia decreased. These results suggest that SIM NPs can reduce neuroinflammatory responses after SAH by activating the Nrf2 pathway, reducing the activation of the STING pathway, and reducing the polarization of M1‐type glial cells (Figure 6K).

### 3.6 Effect of SIM NPs on Long‐Term Neurological Function and Hippocampus Injury

**Figure 7:**
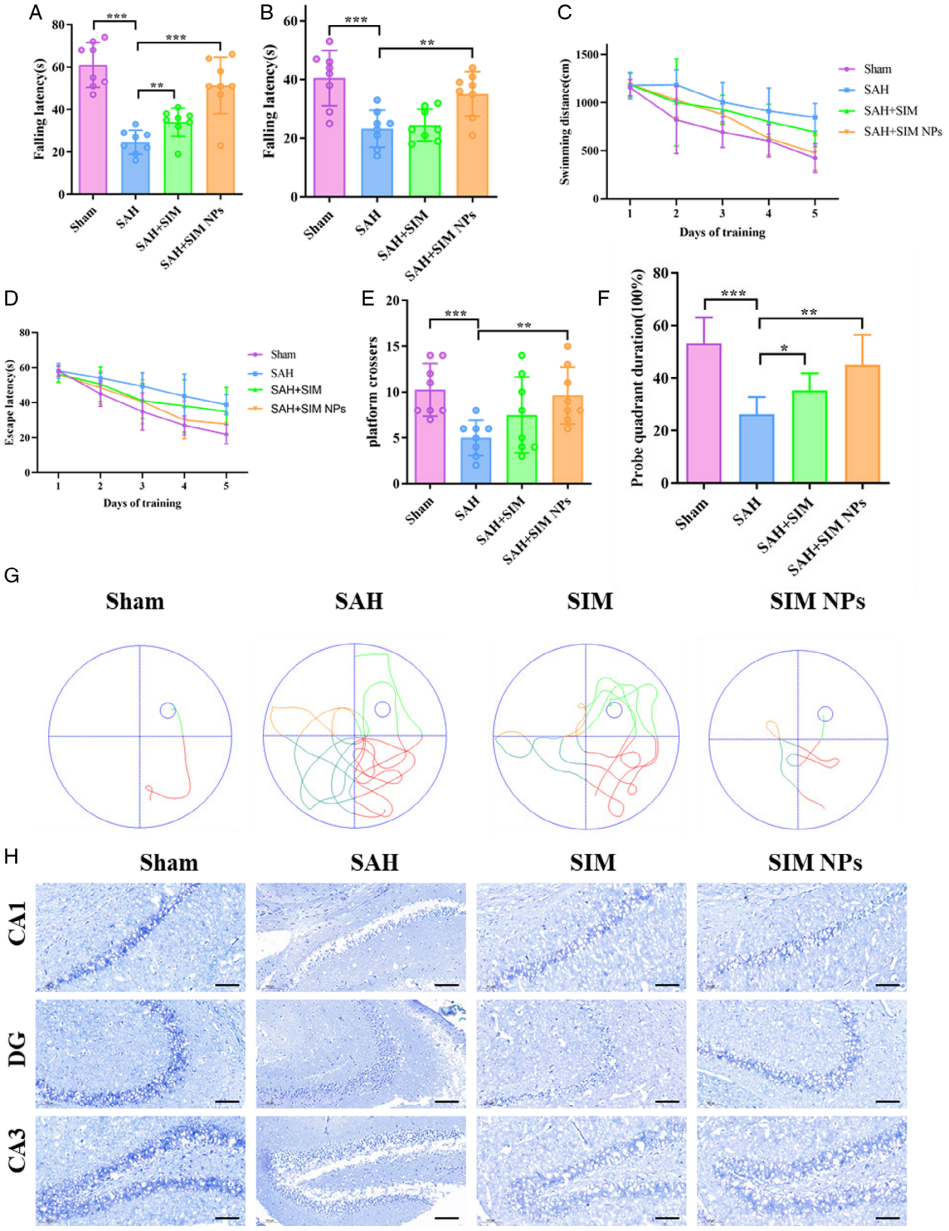
Effect of SIM NPs on persistent neurological function and hippocampus injury. A,B) Fall latency of mice in the Rotarod test at initial speeds of 5 rpm (A) and 10 rpm(B) (*n* = 8). C–F) Swimming distance, platform crossover times, probe quadrant duration, and escape latency of MWM (*n* = 8). *P < 0.05; **P < 0.01; ***P < 0.001. G) Representative swimming trajectories when training. H) Representative images showing Nissl staining of CA1 CA3 and DG regions. Scale bar = 100 μm.

As shown in Figure 1 experiment 4, we further investigate the long‐term effects of SIM NPs on neurofunctional deficits in SAH model mice. After successfully inducing the model, 0.1 mg g^−1^ of SIM NPs was administered at 0, 1, 3, 7, 14, and 21 days. MWM training was conducted from day 22, MWM testing and Rotarod assays were performed on day 27, and the mice were sacrificed on day 28 for histological examination. Rotarod assays were performed using a starting speed of either 5 or 10 rpm. Compared with the sham surgery group, the autumn latency was significantly shortened in the SAH group, while the autumn latency was significantly prolonged in the group treated with SIM NPs, indicating that SIM NPs can effectively counteract SAH‐induced neurofunctional deficits (**Figure** **7**A,B). MWM results showed that differences in the first‐day swimming distance and escape latency were negligible, indicating that the baseline swimming ability of each group was similar (Figure 7C,D). However, compared with the sham surgery group, from day 2 to day 6, SAH mice exhibited increased escape latencies and swimming distances, while the number of platform crossings decreased (Figure 7C–E). This pattern was exemplified in their unique swimming paths (Figure 7G). During the phase of removing the underwater circular platform, the SAH group exhibited reduced exploration in the quadrant containing the platform (Figure 7F). This suggests significant learning and memory deficits caused by hippocampal neuron damage. From day 3 to day 5, SIM NP treatment significantly improved performance by shortening escape latencies, reducing swimming distances, increasing platform crossings, and prolonging exploration time in the platform quadrant. These findings emphasize the therapeutic potential of SIM NPs in mitigating persistent hippocampal damage caused by SAH.

Subsequently, Nissl staining was performed on brain tissue. Compared with the sham surgery group, the number of intact neurons in the DG, CA1, and CA3 regions was significantly reduced in the SAH group. SIM treatment failed to restore neuron counts to normal levels (Figure 7H). In contrast, SIM NPs exhibited a significant surge in the number of surviving neurons. These findings ultimately indicate that SIM NPs can improve long‐term neurofunctional deficits by inhibiting neuroinflammation following SAH and minimizing hippocampal neuron damage.

## 4 Discussion

The investigation into the therapeutic efficacy of self‐assembled SIM NPs for the treatment of SAH reveals promising advances in neuroprotective strategies. The primary contribution of this study is the demonstration of SIM NPs’ ability to attenuate EBI and mitigate neuroinflammation via modulation of the Nrf2/STING signaling pathway. These findings underscore the critical role of targeted NP delivery systems in overcoming the blood–brain barrier, a longstanding obstacle in the treatment of neurological conditions.

The utilization of SIM, a compound with established anti‐inflammatory and antioxidant properties, in the form of NPs, represents a significant innovation in the field of neurotherapeutics. The methodology employed in synthesizing these NPs ensures their effective penetration into the brain tissue, enabling the direct modulation of inflammatory pathways involved in EBI. This approach not only highlights the potential of natural compounds in medical applications but also opens new avenues for the development of nanotechnology‐based therapies for cerebrovascular diseases.

Despite the encouraging outcomes, several challenges and questions remain unaddressed. The precise mechanisms through which SIM NPs exert their neuroprotective effects need further elucidation. While the inhibition of the STING pathway and the promotion of Nrf2 signaling have been identified as key factors in reducing neuroinflammation and neuronal damage, the downstream effects of these modulations on cellular and molecular levels within the brain's microenvironment remain to be fully explored. Additionally, the long‐term safety and efficacy of SIM NPs administration, as well as the potential for off‐target effects, warrant comprehensive investigation in future studies.

Another critical area for future research is the optimization of NP design and delivery. The current study has successfully identified a NP size that optimizes brain delivery and therapeutic efficacy; however, exploring other aspects of NP design, such as surface charge, shape, and the potential for targeted delivery to specific cell types within the brain, could further enhance the therapeutic potential of SIM NPs. Moreover, investigating the interaction of SIM NPs with the brain's immune system, including the blood–brain barrier's role in modulating immune responses, could provide deeper insights into the mechanisms of action and improve the specificity and efficiency of treatment.

## 5 Conclusion

In summary, we synthesized self‐assembled SIM NPs with SIL as the main component, and controlled their size to effectively target and transport them to the SAH site. We further demonstrate that SIM NPs can reduce EBI after SAH by inhibiting the Nrf2/STING pathway, inhibiting neuroinflammation, and inhibiting M1 polarization of microglia.

## Ethics Approval Statement

The reason for using animals is that research requires them to develop new therapies for treating diseases. All mice were purchased from Hunan Slake Experimental Animal Co., Ltd. The mice were kept at a constant temperature under a 12 h dark light cycle and were fed freely with water and standard laboratory feed. Conduct the experiment after one week of adaptation. Anesthetize mice with isoflurane. Gently insert the guided intubation into the trachea in a noninvasive manner to minimize the pain of the mice. Mice were euthanized by inhaling excessive amounts of isoflurane. The Committee of Animal Care and Use of Renmin Hospital of Wuhan University approved all experiments with animals in this study (WDRY20210305A). All methods are reported in accordance with ARRIVE guidelines.

## Conflict of Interest

The authors declare no conflict of interest.

## Author Contributions

**Yong Li**: conceptualization; methodology, software; validation; data curation; writing—original draft. **Youdong Zhou**: software; validation; investigation. **Yinqiu Tan**: conceptualization; supervision; project administration. **Gang Deng**: conceptualization; resources; writing—review and editing; funding acquisition.

## Supporting information

Supplementary Material

## Data Availability

The data that support the findings of this study are available from the corresponding author upon reasonable request.
