# Supplementary material for: Anti‐Inflammatory Treatment of Subarachnoid Hemorrhage by Self‐Assembled Silymarin Nanoparticles
Source: Small Sci. 2025 Feb 3;5(4):2400322. doi: 10.1002/smsc.202400322 (PMC12244996; doi:10.1002/smsc.202400322)
Supplement: Supplementary file 1 — Supplementary Material [file SMSC-5-2400322-s001.pdf]

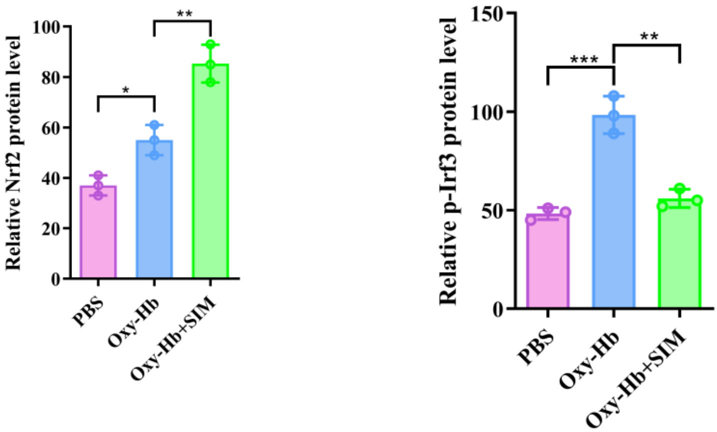

Figure S1. Quantification analysis of Nrf2, STING and p-Irf3 in BV2 cells after 20μg/ml silymarin treatment.

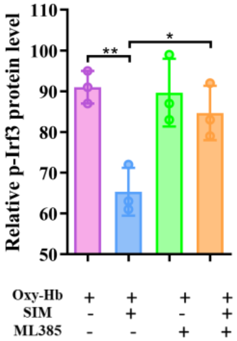

Figure S2. Quantification analysis of STING and p-Irf3 in BV2 cells after 20μg/ml silymarin and ML385 treatment.

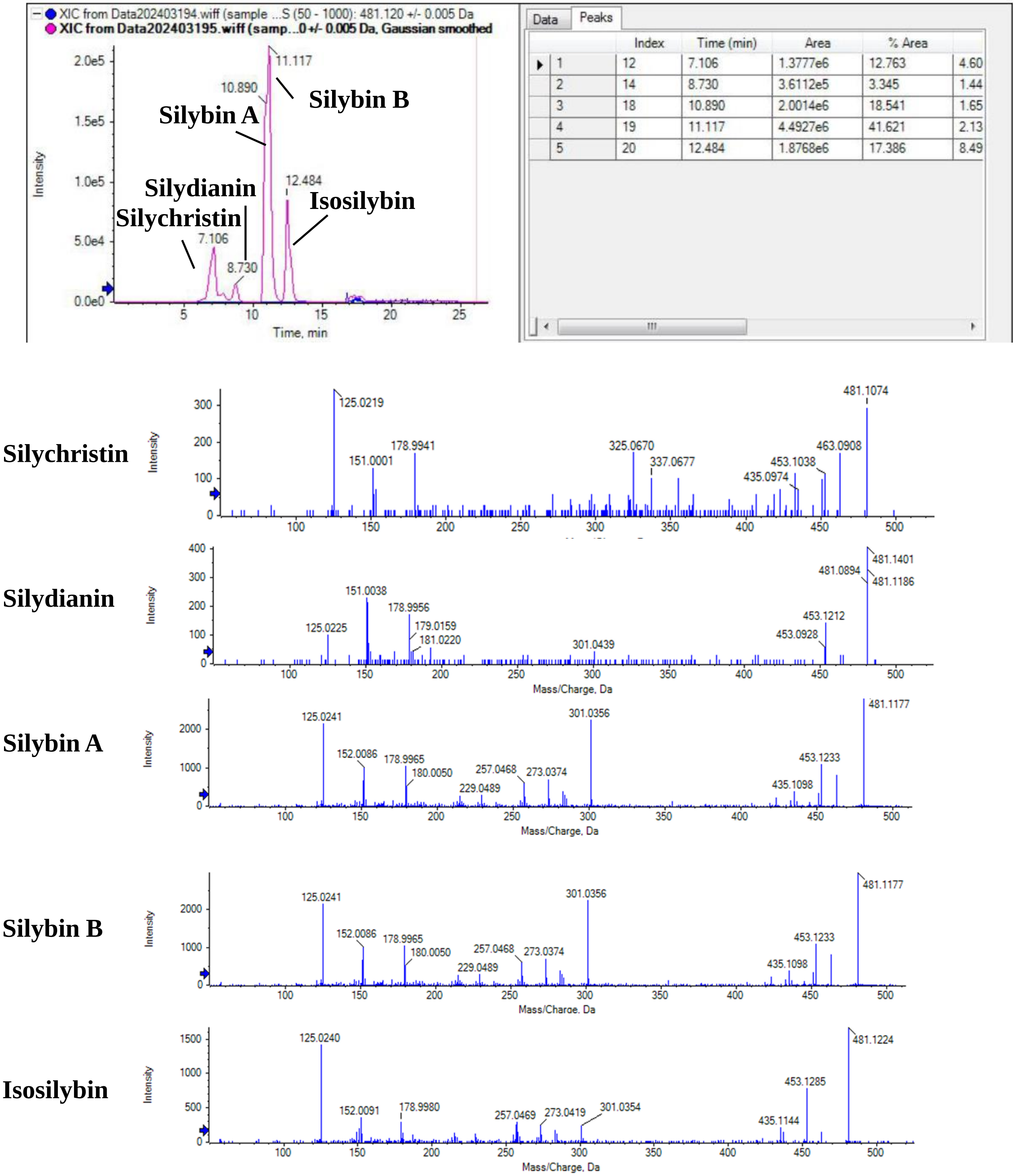

	Index	Time (min)	Area	% Area	
1	12	7.106	1.3777e6	12.763	4.60
2	14	8.730	3.6112e5	3.345	1.44
3	18	10.890	2.0014e6	18.541	1.65
4	19	11.117	4.4927e6	41.621	2.13
5	20	12.484	1.8768e6	17.386	8.49

Figure S3. LC-MS analysis showed the Composition of SIM NPs.

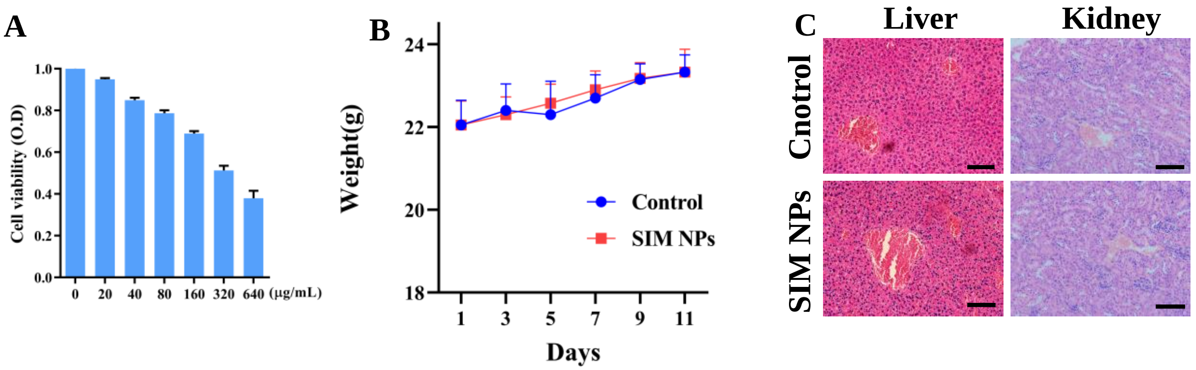

Figure S4. The drug safety of SIM NPs. (A) CCK-8 test for measuring the effect of UA on the proliferation of BV2 cells. (B) Body weight the mice with different treatment. (C) HE staining of liver, kidney, heart and spleen of the mice with different treatment. Scale bar=50μm.
